# Detection of *Deltacoronavirus* in environmental fecal samples from seabirds in the Saint Peter and Saint Paul Archipelago, central equatorial Atlantic Ocean

**DOI:** 10.1371/journal.pone.0314842

**Published:** 2025-06-03

**Authors:** Fernanda Gomes, Alexandre Freitas da Silva, Tatiana Prado, Marina Galvão Bueno, Luciana Appolinario, Patrícia Soares Flores, Paola Cristina Resende, Marilda Siqueira, Leonardo Corrêa, Martha Brandão, Jose Reck, Gabriel Luz Wallau, Maria Ogrzewalska

**Affiliations:** 1 Laboratório de Vírus Respiratórios, Exantemáticos, Entéricos e Emergências Virais, Instituto Oswaldo Cruz, Fundação Oswaldo Cruz, Rio de Janeiro, RJ, Brasil,; 2 Departamento de Entomologia, Instituto Aggeu Magalhães, Fundação Oswaldo Cruz, Recife, PE, Brasil,; 3 Núcleo de Bioinformática, Instituto Aggeu Magalhães, Fundação Oswaldo Cruz, Recife, PE, Brasil,; 4 Laboratório de Virologia Comparada e Ambiental, Instituto Oswaldo Cruz, Fundação Oswaldo Cruz, Rio de Janeiro, RJ, Brasil,; 5 Plataforma Institucional de Biodiversidade e Saúde da Vida Silvestre, Fundação Oswaldo Cruz, Rio de Janeiro, RJ, Brasil,; 6 Instituto de Pesquisas Veterinárias Desidério Finamor, Eldorado do Sul, RS, Brasil,; 7 Universidade Federal Santa Maria, Camobi, Santa Maria - RS, Brasil; Cairo University Faculty of Veterinary Medicine, EGYPT

## Abstract

This study investigates the presence of avian coronaviruses (CoVs), Avian influenza viruses (AIVs), and Avian rotaviruses group A (AvRVs) in seabird populations inhabiting the Saint Peter and Saint Paul Archipelago (SPSPA), isolated and remote oceanic islands situated in the equatorial region of the Atlantic Ocean. In July 2022, 95 environmental fecal samples were collected from seabird colonies and screened for these viruses by quantitative one-step real-time RT-PCR (AIVs and AvRVs), by the conventional pancoronavirus RT-PCR protocols and metatranscriptomics of a positive sample. Four environmental samples tested positive for CoVs. Avian AIVs and AvRVs were not detected. Phylogenetic analyses revealed CoVs closely related to avian deltacoronaviruses previously identified in waterbirds from Asia and Australia. We could not recover the CoV by metatranscriptomics but we recovered a single viral contig of an avian enterovirus. The findings contribute valuable insights into virus dynamics among seabird populations, laying the groundwork for future investigations in this field.

## Introduction

Seabirds encompass a diverse array of species, spanning at least six avian orders: Sphenisciformes, Procellariiformes, Pelecaniformes, Suliformes, Phaethontiformes, and Charadriiformes. A unifying characteristic among these groups is their reliance on marine environments for feeding [1]. Most seabirds exhibit colonial breeding behavior, forming large aggregations during the breeding season. While they demonstrate strong fidelity to breeding colonies, seabirds also undertake extensive migrations outside the breeding period, foraging and exploring potential future nesting sites [1]. This behavior highlights the potential role of seabirds as important hosts in the dispersal of infectious agents across diverse ecosystems [2–5].

Research on seabird parasites (organisms such as ectoparasites and protozoa that live on or within a host, which can also be pathogens) and pathogens (disease-causing agents, including bacteria and viruses) has suggested a diverse range of infecting organisms associated with these birds [3,6–11]. Among viruses associated with seabirds, coronaviruses (CoVs), Avian influenza viruses (AIVs) and Avian rotaviruses (AvRVs) warrant special attention due to their potential to cause significant morbidity and, in some cases, mass mortality among both wild and domestic birds. The AIVs are often considered the most significant. While these viruses typically circulate among wild birds without causing noticeable illness, some bird species can develop disease [12]. Some strains can become highly pathogenic, causing severe disease and high mortality rates in poultry and wildlife. Notably, the highly pathogenic AIV (HPAI A/H5N1 clade 2.3.4.4b) has caused massive mortality in marine fauna across South American countries, including Argentina, Bolivia, Brazil, Chile, Colombia, Ecuador, Paraguay, Peru, Uruguay, and Venezuela, since October 2022 [13–19]. For instance, more than 100,000 wild birds were reported to have been killed by this pathogen in Peru alone [20] and least 24,000 sea lions died in Peru, Chile, Argentina, Uruguay, and Brazil between January–October 2023 [21]. The virus has demonstrated sustained transmission among mammals in various environments, such as European fur farms, U.S. dairy cattle, and South American marine mammals, raising concerns about the potential risk to humans [22].

CoVs cause a variety of diseases in animals, including upper and lower respiratory infections, gastroenteritis, and central nervous system disorders [23]. In birds, CoVs are widely distributed, causing diseases ranging from mild respiratory and gastrointestinal infections to severe outbreaks in poultry. For instance, *Avian coronavirus*-ACoV infectious bronchitis virus (IBV), is known to affect domestic chickens, leading to respiratory issues, reduced egg production, and, in severe cases, mortality [24]. Besides chickens, viruses from this genus also infect other species, including turkeys, pheasants, and guinea fowl, where they are likewise linked to disease [25]. However, in most cases, wild birds remain asymptomatic, although they can act as hosts with potential implications for zoonotic transmission [26–28].

AvRVs infect the intestinal tract of birds, causing diarrhea and enteritis, particularly in young birds, which often suffer high mortality rates. They affect various domestic avian species, including turkeys, chickens, and pheasants, with clinical signs such as dehydration, stunting, depression, and distended intestines varying by strain [29]. AvRVs have also been detected in wild birds, with Rotavirus A (RVA) identified in families such as Turdidae, Fringillidae, Thraupidae, Psittacidae, and Ramphastidae, some of which exhibited diarrhea [30]. Asymptomatic RVA infections have been reported in migratory birds like velvet scoter *Melanitta fusca* [31]. Research on AvRVs in seabirds, their co-occurrence with other viruses, and the genotypes involved across host species remains limited.

The potential for these viruses to cause mass mortality is particularly concerning at the population level, as it can lead to long-term declines in seabird populations, disrupting the dynamics of local ecosystems. This is particularly true for species with small, localized populations, such as those in the Saint Peter and Saint Paul Archipelago (SPSPA) where even a modest outbreak could significantly impact the breeding success of the resident seabirds.

**Fig 1:**
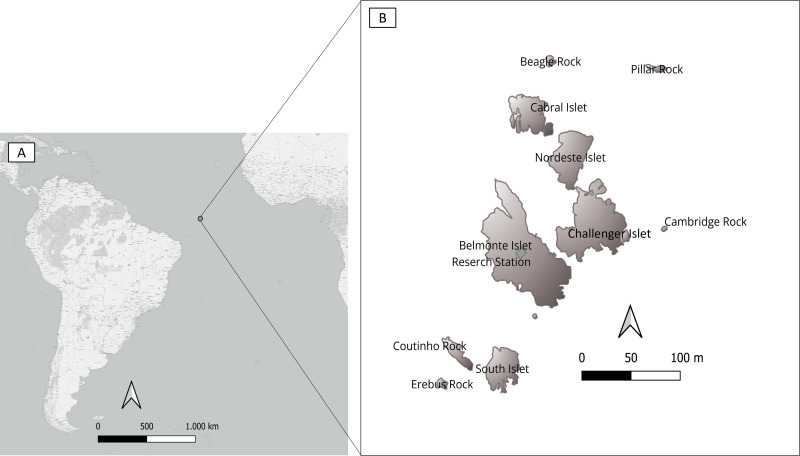
Location of Saint Peter and Saint Paul Archipelago, central equatorial Atlantic Ocean (A). Saint Peter and Saint Paul Archipelago with main islets, rocks, and research station (B). The map was created using the free software QGIS [33]. The islands were digitized by the authors, and free shapefiles of the map were obtained from the Instituto Brasileiro de Geografia e Estatística (IBGE) [Shapefile of the map in the scale 1:250,000, available from: https://portaldemapas.ibge.gov.br/portal.php#homepage].

The SPSPA is a small group of rocky islets and reefs of volcanic origin, located in the middle of the Atlantic Ocean (Fig 1). The archipelago is home to three seabird species that breed locally in high densities: black noddy *Anous minutus*, brown noddy *Anous stolidus* (Charadriiformes, Laridae) and brown booby *Sula leucogaster* (Suliformes, Sulidae). Nonetheless, the SPSPA is sporadically visited by various seabird species such as masked booby *Sula dactylatra*, red-footed booby *Sula sula*, magnificent frigatebird *Fregata magnificens* (Suliformes, Fregatidae), and sooty tern *Onychoprion fuscatus* (Charadriiformes, Laridae). Other migratory waterbirds occasionally appear including species such as herons - cattle egret *Bubulcus ibis* (Pelecaniformes: Ardeidae), little egret *Egretta gularis*, and white egret *Egretta garzetta* - as well as the lesser moorhen *Paragallinula angulata* (Gruiformes: Rallidae). Even birds of prey, such as the common kestrel *Falco tinnunculus* (Falconiformes: Falconidae), and shorebirds like the turnstone *Arenaria interpres* and yellowlegs *Tringa flavipes* (Charadriiformes: Scolopacidae), have been recorded occasionally [32]. The interactions between resident and migratory birds in this environment may facilitate inter-species viral transmission.

No previous study has been conducted at this field site to investigate the presence of viruses. To address this research gap, we conducted a study to collect environmental samples from seabirds to test for the presence of AIVs, CoVs and group A AvRVs. By examining these samples, we aimed to gain valuable insights into the potential role of seabirds as hosts of these viruses in this unique and isolated ecosystem.

## Materials and methods

### Study area and sample collection

The SPSPA is part of the territorial waters of Brazil and is situated approximately 1010 km off the northeastern coast of Brazil (00°55’10“N, 29°20’33”W). The rocky formation of the Archipelago covers an area of approximately 17 000 m², with a maximum altitude of 18 m. The SPSPA consists of six main islets: Cabral, Nordeste, Challenger, Belmonte, South and Coutinho (Fig 1). The archipelago is not inhabited and lacks domestic or wild mammals. However, the largest island, Belmonte Island, hosts a Brazilian Navy research station.

**Fig 2:**
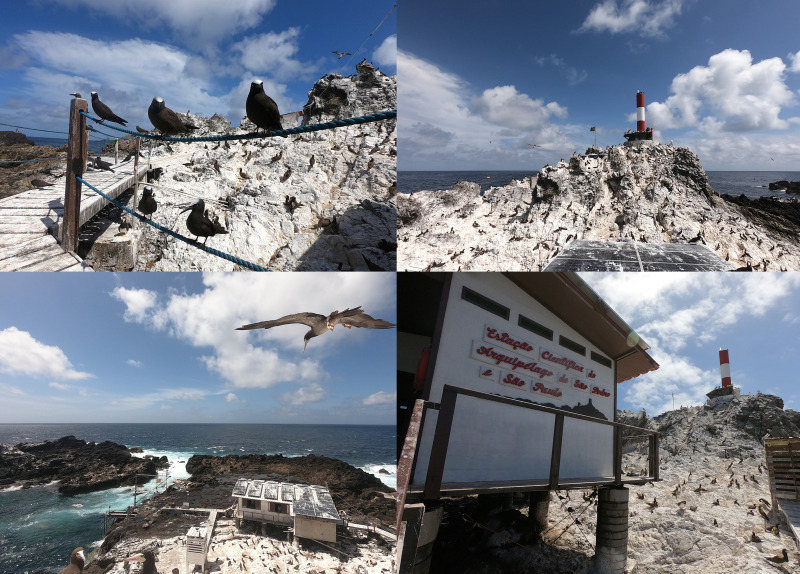
Images from the Belmonte Island show high densities of seabirds and the research station covered in guano. July 2022, Saint Peter and Saint Paul Archipelago, central equatorial Atlantic Ocean. Photos by M. Brandao.

As shown in Fig 2, *S. leucogaster*, *A. minutus*, and *A. stolidus*, along with their nests, are dispersed throughout the area, often near human activity at the station. Convenience fresh environmental fecal samples were collected over three consecutive days in July 2022 on Belmonte Islet. Samples were collected from the surrounding area and the surface of the solar panels on the station’s roof (which were cleaned twice daily due to the large accumulation of excrement of seabirds), while carefully observing the defecation behavior of each bird. Fresh fecal droppings were collected using sterile Dacron swabs, which were promptly placed into sterile tubes containing 1 mL of RNAlater™ Stabilization Solution (Invitrogen™) for preservation. The samples were kept at room temperature for seven days before being frozen at -20°C for further analysis. Basic personal protective equipment (PPE), such as gloves and N95 masks, was used during the collection process.

### Viral RNA extraction

Clarified fecal suspensions (20%, wt/vol) were prepared with phosphate-buffered saline (PBS) as previously described [34]. Viral RNA was extracted using a QIAamp viral RNA mini kit (Qiagen, CA, USA) according to the manufacturer’s instructions. For each RNA extraction procedure, RNase/DNase-free water was used as a negative control.

### Avian Influenza virus (AIVs) and group A avian rotavirus (AvRVA) screening by quantitative one-step real-time RT-PCR

The AIVs and AvRVA were screened using TaqMan based quantitative one-step real-time RT-PCR designs. All reactions were performed using the SuperScript III Platinum one-step quantitative RT-PCR (qRT-PCR) kit (Thermo Fisher Scientific) according to the protocol established by the Collaborative Influenza Center, Centers for Disease Control and Prevention, Atlanta, GA for detection of all influenza A [35] and [30,36] for detection of all rotavirus A. The samples with Cycle threshold (Ct) < 40 were considered positive.

### Coronavirus (CoVs) screening by conventional pancoronavirus RT-PCR

All the samples were also subjected to pancoronavirus RT-PCR targeting the RNA-dependent RNA polymerase (*RdRp*) gene as described previously [26]. Briefly, cDNA was obtained and amplified in a first-round PCR (RdRpS1 5´-GGKTGGGAYTAYCCKAARTG-3’, RdRpR1 5’-TGYTGTSWRCARAAYTCRTG-3’) using One-Step RT-PCR Enzyme MixKit (Qiagen) with the total expected amplicon size of 602 base pairs (bp). Following, the nested PCR were conducted using Phusion RT-PCR Enzyme Mix kit (Sigma-Aldrich), primers Bat1F 5’-GGTTGGGACTATCCTAAGTGTGA-3’ and Bat1R 5’-CCATCATCAGATAGAATCATCAT-3’ and 1 uL of the amplified product as a template were used. *RdRp* amplicons (~440 bp) were visualized on 1.5% agarose gels with SYBR™ Safe DNA Gel Stain (Thermo Fisher Scientific).

### Sanger sequencing

*RdRp* amplicons were purified using the QIAquick Gel Extraction Kit (Qiagen) following the manufacturer’s recommendation. The Sanger sequencing reaction was prepared using BigDye Terminator v3.1 Cycle Sequencing Kit (Life Technologies) with primers Bat1F and Bat1R. The sequencing of both strands was performed using the ABI 3730 DNA Analyzer (Applied Biosystems).

### Gene assembly and phylogenetic analysis

The reads generated by Sanger sequencing were evaluated and assembled using Sequencher 5.1 (GeneCodes). The obtained consensus was checked by the chromatogram analysis and the final consensus was uploaded at the National Center for Biotechnology Information (NCBI) GenBank database. To enhance our understanding of the phylogenetic relationships among the sequences generated in this study we performed homologous sequence retrieval from the non-redundant NCBI database based on blastn of all partial *RdRp* obtained in this study. The dataset for analysis was compiled to include: (i) sequences determined in this work (n = 3), (ii) the most similar sequences from GenBank to those from this work, obtained from a BLAST analysis (hits with the highest score, identity > 70%, and coverage > 60%, excluding duplicates, n = 54), (iii) reference sequences for all three subgenera of the genus *Deltacoronavirus* as defined by the ICTV (n = 7), and (iv) reference sequences from members of the genus *Gammacoronavirus* (n = 6) for rooting the tree [37]. Nucleotide alignments were performed with MAFFT v7.0 online (https://mafft.cbrc.jp/alignment/server/index.html). (https://ictv.global/report/chapter/coronaviridae/coronaviridae). The best nucleotide substitution model was defined by MEGA X and used for the reconstruction of the maximum likelihood (ML) phylogenetic tree of the partial *RdRp* gene [38] with bootstrap values obtained with 500 replicates. The ML tree was annotated in FigTree v.1.4.4 (http://tree.bio.ed.ac.uk/software/figtree/).

### Metatranscriptomic sequencing

One CoV positive sample (A22801) that reached enough RNA quantity and quality was processed to metatranscriptomic sequencing aiming to characterize their viral content. The previously extracted RNA was treated with Ambion® TURBO DNA-free™ Kit (Invitrogen) following the manufacturer’s instructions to remove residual genomic DNA. Host rRNA was depleted using the Illumina Ribo-Zero Plus rRNA Depletion Kit (Illumina, San Diego, CA, USA). After rRNA depletion, the remaining RNA was converted into cDNA and prepared for library construction using the Illumina® DNA Prep Kit. The libraries were sequenced on the Illumina® NextSeq platform (Illumina, San Diego, CA, USA) using a NextSeq 1000/2000 P2 cartridge employing a paired-end approach. The raw sequence data were submitted to the NCBI Sequence Read Archive (SRA) database and are available under the project number PRJNA1183304.

After sequencing, the raw reads were first analyzed on fastp v0.23.2 and the low-quality reads were trimmed with a cutoff of 20 for Phred score and a minimum read length of 36 bp. Trimmed reads were assembled using a *de novo* approach on metaSPAdes v3.15.5 in default mode [39]. The assembled contigs were submitted to a pairwise-alignment analysis employing Diamond blastx [40] against a custom dataset constructed based on all viral proteins assigned to taxonomy tag (txid10239) from NCBI plus *RdRp* sequence databases such as NeoRdRp [41], PalmDB [42] and RdRp-Scan [43] as performed previously [44]. Briefly, the sequences showing the best hits on the viral database were submitted to a second Diamond blastx analysis against the non-redundant (NR) database from NCBI to remove false negative hits. A BLASTn [45] analysis was also performed to identify the best nucleotide hits based on the NT database from NCBI. The completeness of viral sequences were assessed on ViralComplete [46] tool that classified them into full-length or partial according to their best hit with NCBI RefSeq genomes.

All applicable national and institutional guidelines for sampling, care and experimental use of organisms for the study were followed and all necessary approvals were obtained such as Permits of Instituto Chico Mendes de Conservação da Biodiversidade (SisBio 84084–1) and the registration of the project in the Sistema Nacional de Gestão do Patrimônio Genético e do Conhecimento Tradicional Associado (A21FE59). All permits are available on request.

## Results

**Fig 3:**
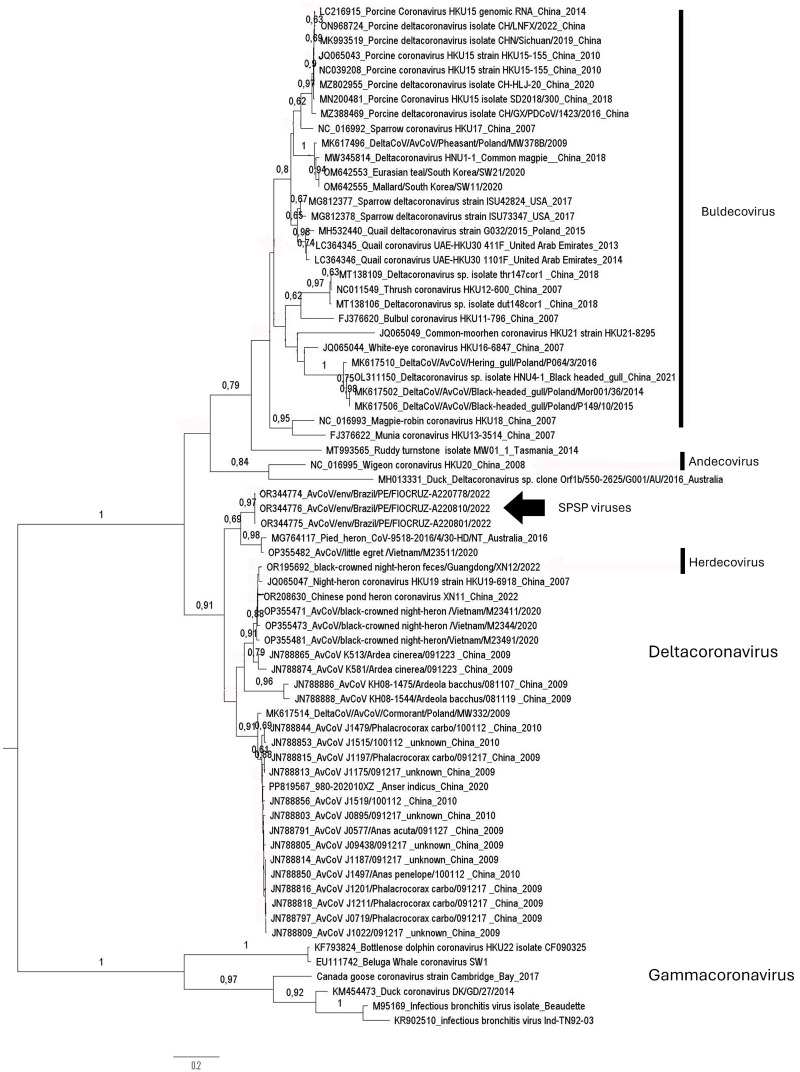
A Maximum Likelihood phylogenetic tree, focusing on deltacoronaviruses, was reconstructed using the best-fit nucleotide substitution model, General Time Reversible (GTR + G). This tree highlights the positions of viruses identified in the present study (arrow). The final dataset comprised 70 partial genomes, specifically 360 base pairs of the *RdRp* gene.

During three consecutive days of collecting fresh droppings, only three bird’s species were observed on the islands: *A. minutus*, *A. stolidus* and *S. leucogaster*. A total of 95 environmental fecal samples were collected and tested for selected viruses. Four samples tested positive for CoVs targeting *RdRp* genome region. After the *RdRp* nucleotide Sanger sequencing of the positive samples, it was possible to obtain fragments of genomes from three of them. The three *RdRp* fragment sequences are available at GenBank database as AvCoV/env/Brazil/PE/FIOCRUZ-A220778/2022 (OR344774), AvCoV/env/Brazil/PE/FIOCRUZ-A220801/2022 (OR344775) and AvCoV/env/Brazil/PE/FIOCRUZ-A220810/2022 (OR344776). Obtained viruses were 99.5–99.7% identical between themselves and the closest identity of 97% (218/225 bp) was observed with avian *Deltacoronavirus* found in swan goose *Anser cygnoides* (Anseriformes, Anatidae) from southern Brazil (KU321643, KU321644) in 2013. The phylogenetic reconstruction of *RdRp* gene fragment revealed the three CoVs detected in the present study cluster in the unnamed clade of deltacoronaviruses, with a sister clade of viruses detected previously in herons (Pelecaniformes, Ardeidae) from Vietnam and Australia in 2020 and 2016 respectively (Fig 3). The sequences from Brazil were not included in phylogenetic analyses because the deposited in GenBank RdRp gene fragments were only 225 bp long, with coverage of 54% with our sequences.

In addition to our previous analysis, we further investigated one sample positive for CoV (A22801) using a metatranscriptomic approach. The sequencing yielded 57.28 million raw reads and 50.72 million quality-filtered reads were assembled. Diamond analysis revealed the presence of three viral Enterovirus contigs with size ranging from 1,494–3,352 bp but no *Deltacoronavirus* contigs were detected. *Enterovirus* contigs showed high identity with human *Enterovirus 99*, with amino acid identities ranging from 52% to 98.5 (ABM54518.1 and QRG33086.1) and nucleotide analysis identities ranging from 82.7% to 84.06 (EF015008.1 and EF015009.1). Avian AIVs and AvRVs were not detected in all tested samples.

## Discussion

Marine bird health in Brazil remains largely understudied, with limited research on viruses and antimicrobial resistance [9,47–49]. Few studies have focused on these aspects in Brazilian seabirds, with the primary challenge being the remote and inaccessible nature of many breeding sites. The ASPSP represents one of the most isolated ecosystems in the Atlantic Ocean, making it a unique and valuable location for investigating the potential health risks posed by viral pathogens in marine birds. This study aimed to assess the presence of three significant viruses in fecal samples from marine birds on the ASPSP. While all samples were negative for AIVs and AvRVs, four samples tested positive for *Deltacoronavirus*.

Most deltacoronaviruses have been found in birds, the only exception being porcine coronavirus HKU15, which has been associated with fatal outbreaks in pigs and occasionally infecting humans [50,51]. Deltacoronaviruses have been identified across 15 bird orders, involving over 100 species of wild birds. They are most frequently observed in waterfowl and shorebirds, particularly within the Charadriiformes and Anseriformes orders. In most cases, these viruses do not cause noticeable signs of disease in the infected birds [26,52,53]. Although wild birds may often carry CoVs asymptomatically, the detection of these viruses is significant as it may indicate potential transmission risks to other species, including domestic birds and, in some cases, mammals. This is particularly concerning due to their potential to mutate and cross the species barrier, potentially leading to outbreaks that could affect animal populations and human health [23,54–56].

Three subgenera can be distinguished within the *Deltacoronavirus* genus, namely *Andecovirus* (one species), *Buldecovirus* (five species) and *Herdecovirus* (one species). However, we were not able to classify the viruses found in the present study into any of these three subgenera. We acknowledge the limitations of our work, as we were unable to recover the full genome of the viruses obtained and conduct more detailed analyses. The viruses sequences generated in this study, as well as many others available in GenBank, present only small genome fragments and only viruses for which a complete genome sequence are available might be considered for taxonomy to define possible new subgenera [37].

Previous research has shown that wild aquatic birds of the order Anseriformes and Charadriiformes are the natural host of AIVs [57]. However, our analysis did not reveal the presence of influenza virus in any of the collected environmental samples. This outcome contrasts with previous studies reporting positive detections of AIVs in similar habitats [58]. The absence of influenza virus detection in the collected environmental samples could be attributed to several factors and does not necessarily imply an absence of the virus itself. The short duration of the sampling period, which was limited to only three days, might have influenced the absence of detection of influenza viruses. Our brief three-day sampling window might not have encompassed the entirety of the transmission dynamics of the virus through time, potentially missing windows of active viral shedding. Additionally, a limited number of samples was collected and tested.

Just like the absence of influenza virus infection, the lack of detection of avian RVAs in the feces of the birds can be explained by short sampling period and the possibility of prior acquisition of immunity, considering the long-life span of the birds. However, it could be assumed that there is no circulation of avian RVAs in the bird populations that inhabit the island, in the period sampled, since fresh feces were collected and the high capacity of these viruses to survive in the environment. *Rotavirus* are non-enveloped viruses, which make them stable and infective for a long period in the environment without suffering degradation and inactivation. Similar to other enteric viruses, all RVAs are resistant to chemical and physical agents in the environment [59]. RVAs are not well studied among wild-living birds including seabirds, however, some recent studies suggest that the genetic diversity of avian RVAs is greater than previously recognized, infections occur in a wide spectrum of bird species and that migratory birds may contribute to the global spread of these viruses [31,60,61].

Research on the migratory behavior of *Anous* and *S. leucogaster* at the SPSPA remains limited. However, a few studies suggest that these populations are not migratory or partially migratory within the region. This conclusion is based on observations that their population sizes do not exhibit significant seasonal variations throughout the year, nor is there evidence of substantial migrations involving large portions of the population [62]. *Anous* breed at the ASPSP between March and September [63], while *S. leucogaster* breed throughout the year, showing the absence of seasonality in nesting [62,63]. Other species appear on the ASPSP occasionally, however long‐distance Nearctic–Neotropical migrant species, especially waders (Charadriiformes), are of particular concern as they are primary hosts of influenza viruses. These birds, such as *A. interpres* and *T. flavipes*, are known to migrate from their wintering grounds in South America, including coastal regions along the Atlantic, to breeding areas in the Arctic and other temperate zones [64–66]. Their migratory patterns occasionally bring them through remote sites like the ASPSP, where they could introduce the H5N1 virus or other pathogens, posing a risk to resident bird population. However, we consider the exposure to be very low, as the ASPSP is not a primary migratory stopover site where shorebirds gather in high densities, and they are more likely to pass through by chance [64].

The ASPSP holds significant ecological and strategic value and is designated as both an Environmental Protection Area (APA) and a Conservation Unit (UC), under the management of the Chico Mendes Institute for Biodiversity Conservation (ICMBio). Access to the archipelago is tightly controlled to ensure the protection of its marine and terrestrial ecosystems.

While there are no permanent residents, the research station at the ASPSP is regularly occupied by four civilian researchers who rotate every 15 days. The selection process for these researchers includes health-related precautions and specific requirements. They are transported via hired fishing boats from the cities of Natal and Recife as part of a collaboration between SISBIO and various research projects. Additionally, quarterly expeditions, supported by the Brazilian Navy, are carried out to perform maintenance on the station [67]. Researchers at the ASPSP work near marine birds, a common scenario in remote island environments with high seabird densities. The archipelago has designated zones with restricted access to nesting areas, ensuring minimal disturbance to wildlife. While this study did not assess potential transmission risks, the presence of avian viruses in seabird populations globally underscores the importance of biosecurity measures. The use of personal protective equipment (PPE) remains a standard precautionary practice for researchers working in wildlife environments. Further studies are needed to expand knowledge on the virome of seabirds in the ASPSP and their role in viral ecology.

## Conclusion

In conclusion, while our study did not detect AIVs or AvRVAs, the identification of deltacoronaviruses in the fecal samples from seabirds at the ASPSP provides important insights into the viral diversity present in this remote region. Given the isolation of the ASPSP and the migratory patterns of birds, these findings emphasize the need for ongoing surveillance to better understand the circulation of viruses and their potential impact on both wildlife and human health. Future research should focus on expanding the sampling period, increasing the number of samples collected, and utilizing genomic sequencing to further characterize the viruses identified. This would allow for more precise classification and help assess whether these viruses pose any emerging risks to the region.
